# Phase evolution, morphological, optical and electrical properties of femtosecond pulsed laser deposited TiO_2_ thin films

**DOI:** 10.1038/s41598-020-67367-x

**Published:** 2020-06-23

**Authors:** E. Kumi-Barimah, R. Penhale-Jones, A. Salimian, H. Upadhyaya, A. Hasnath, G. Jose

**Affiliations:** 1 School of Chemical and Process Engineering, University of Leeds, Clarendon Road, Leeds, LS2 9JT UK; 2 Department of Engineering, London South Bank University, 103 Borough Road, London, SE1 0AA UK

**Keywords:** Engineering, Materials science, Nanoscience and technology

## Abstract

In this paper, we report anatase and rutile titanium oxide (TiO_2_) nanoparticulate thin films fabricated on silica and Indium Tin Oxide (ITO) substrates using femtosecond pulsed laser deposition (fs-PLD). Depositions were carried-out at substrate temperatures of 25 °C, 400 °C and 600 °C from anatase and rutile phase target materials. Effect of substrate temperature on the surface morphology, microstructural, optical, and electrical properties of these films were systematically investigated by using various range of measurements such as scanning electron microscopy, (SEM), transmission electron microscopy (TEM), X-ray diffraction (XRD), Raman spectroscopy, Ultraviolet–visible-near infrared (UV–Vis–NIR) spectroscopy, and Hall Effect measurements. It is observed that the TiO_2_ thin films surface are predominated with nanoparticulates of diameter less 35 nm, which constitute about ~ 70%; while the optical bandgaps and electrical resistivity decrease with increasing substrate temperature. A mixed-phase (anatase/rutile) TiO_2_ thin film was produced at a substrate temperature of 400 °C when samples are fabricated with anatase and rutile target materials. The results of this study indicate that the structural and crystallinity, optical, and electrical properties can be controlled by varying fs-PLD process parameters to prepare TiO_2_ thin films, which are suitable for applications in photovoltaics, solar cells, and photo-catalysis.

## Introduction

Titanium dioxide, TiO_2_, is among the most studied semiconductor metal oxides in recent years, because of its wide range of applications such as electro-optical devices, self-cleaning and/or antifogging surface coatings, microelectronics, solar cells, photocatalyst, gas sensors, lithium ion batteries, energy harvesting/storage and photovoltaics^1–6^. The TiO_2_ nanoparticles and nanorods have unique and improved properties for designing such devices, which can enhance their performance. Nanoparticle’s size, shape, phase, and morphology play significant role in the optical and electrical properties. For instance, Gratzel et al.^4^ demonstrated low cost and highly efficient dye-sensitized colloidal nanosized TiO_2_ solar cells, with superior performance over the flat layered photoelectrodes. Similarly, Kuang et al.^11^ also synthesised TiO_2_ nanotube arrays with electrochemical anodization technique, for use as a photoanode in the dye-sensitized solar cells. A significant enhancement in photovoltaic performance was observed due to structural arrangement and improved electron transport.

TiO_2_ exists in three different crystalline structures; anatase (trigonal), rutile (tetragonal) and brookite (orthorhombic)^1,7^. All the TiO_2_ polymorphs are low-cost semiconductor materials with fascinating properties, including high transparency, chemical stability, relative hardness and wide optical bandgap range (3.0–3.2 eV)^6,9,10^. The optical and electrical properties depend mainly on the microstructures, nanostructures, crystallinities and surface area of the TiO_2_ nanoparticles, as well as their production method. For instance, TiO_2_ nanoparticles smaller than 20 nm are thermodynamically the stable phase for anatase crystal form^11,12^, whilst those particle with sizes larger than 20 nm demonstrate high stability for rutile crystal form^13^. Amorphous or crystalline TiO_2_ thin films can be fabricated at a very low temperature by the sol–gel method, which would result in low electrons or charge carriers mobilities^14^. TiO_2_ thin films with poor electron mobilities will lead to poor device performance for photocatalytic activities, solar cells, and gas sensing applications. As a result, combining heat treatment with a sol–gel technique is used to enhance electrons charge mobilities and consequently improve the performance of such electronic devices. However, at the elevated temperatures, ranging from 200 to 1,000 °C, the phase transformation of TiO_2_ anatase-to-rutile occurs depending on the fabrication technique^8^. The phase transformation is irreversible. Very recently, great attention has been given to TiO_2_ rutile thin films based on various experimental and theoretical investigations owing to its scientific and practical significance. For instance, Chen et al.^15^ synthesised rutile TiO_2_ nanorods for photocatalytic analysis and then used to fabricate rutile TiO_2_ thin films onto F-doped tin-oxide for dye-sensitized solar cells. They demonstrated that the photocatalytic activity upsurges with an increase of hydrothermal time, while fill factor and energy conversion efficiency were found to be ~ 60% and ~ 3.16% for the dye-sensitized solar cells. Furthermore, the mixed-phase of anatase and rutile TiO_2_ polymorph has a huge advantage for photocatalytic activities and gas sensing device performance since the mixed-phase has improved charge or electron transfer separation and reduces the electron recombination^16–18^. Besides, the electrons transfer in the mixed-phase lead to anatase–rutile interface trapping site, where the electrons migrate from rutile to anatase in the lower energy anatase lattice (Ti^4+^) sites^19^.

TiO_2_ nanoparticulate thin films have been fabricated by numerous deposition methods; among these are filtered arc deposition^20^, solution gelation (sol–gel) process^21^, reactive sputtering method^22^, chemical vapor deposition^23^, metal–organic chemical vapor deposition^24^, and pulsed laser deposition (PLD)^25^. However, PLD is considered as a promising technique to deposit uniform TiO_2_ thin films (anatase–rutile mixed phase or nanoparticle-assembled) because the stoichiometric composition of the thin films deposited is identical to the target material used. This technique has several other advantages which include a fast deposition rate, easy to control film growth, film thickness, nanoparticles size or surface roughness while deposition parameters such as energy, temperature, pressure, target-substrate distance can be adjusted to optimise the fabricating process. There have been numerous studies of TiO_2_ thin films fabricated using nanosecond (ns) PLD^26–28^ in relation to the ablation plasma plume and characteristics of as deposited films, however, femtosecond (fs) PLD bear potential for further investigation. Even though, the ns-PLD is capable of growing nanostructure film, nevertheless, the nanoparticles are over imposed by larger particles in micron range (micro-particulate) which is disadvantageous for designing miniaturize devices for some of the applications mentioned above. In contrast, fs-PLD technique is appealing to produce uniform nanoparticle size with several advantages over ns-PLD as reported by Kumi-Barimah et al.^29^ and Semaltianos et al.^30^. The fs laser materials interaction comprises of ultra-short pulsed length with minimum lattice thermal heating effect, henceforth, the ablation process is quite different from ns-laser. The thin films produce by fs-PLD technique have different morphology, structure and crystallinity depending on the deposition parameters, in particular laser fluence^31^. For example, fractal TiO_2_ nanostructures (not thin films) were fabricated by utilising nonthermal fs-PLD under ambient pressure and temperature^32,33^. They obtained an average nanoparticle diameter below 20 nm, whereas the larger nanoparticles have a diameter above 50 nm. These papers reported that the size of fractal nanostructure distribution depends mainly on the laser fluence and distance between the target-substrate. In addition, these experimental results were also compared to a theoretical model, which confirmed that the fractal nanostructure aggregates are formed through a diffusion mechanism^34^.

In this paper, we report the production of anatase/rutile mixed-phase and rutile TiO_2_ nanoparticulate thin films from anatase and rutile TiO_2_ target materials onto silica and indium tin oxide substrates using fs-PLD technique. The influence of the substrate temperature on the surface morphology, structure, composition, optical and electrical properties of these thin films prepared was systematically studied under various experimental techniques, which have potential for photocatalytic and photovoltaic applications.

## Results and discussion

### Surface morphology and structural characterisation

**Figure 1:**
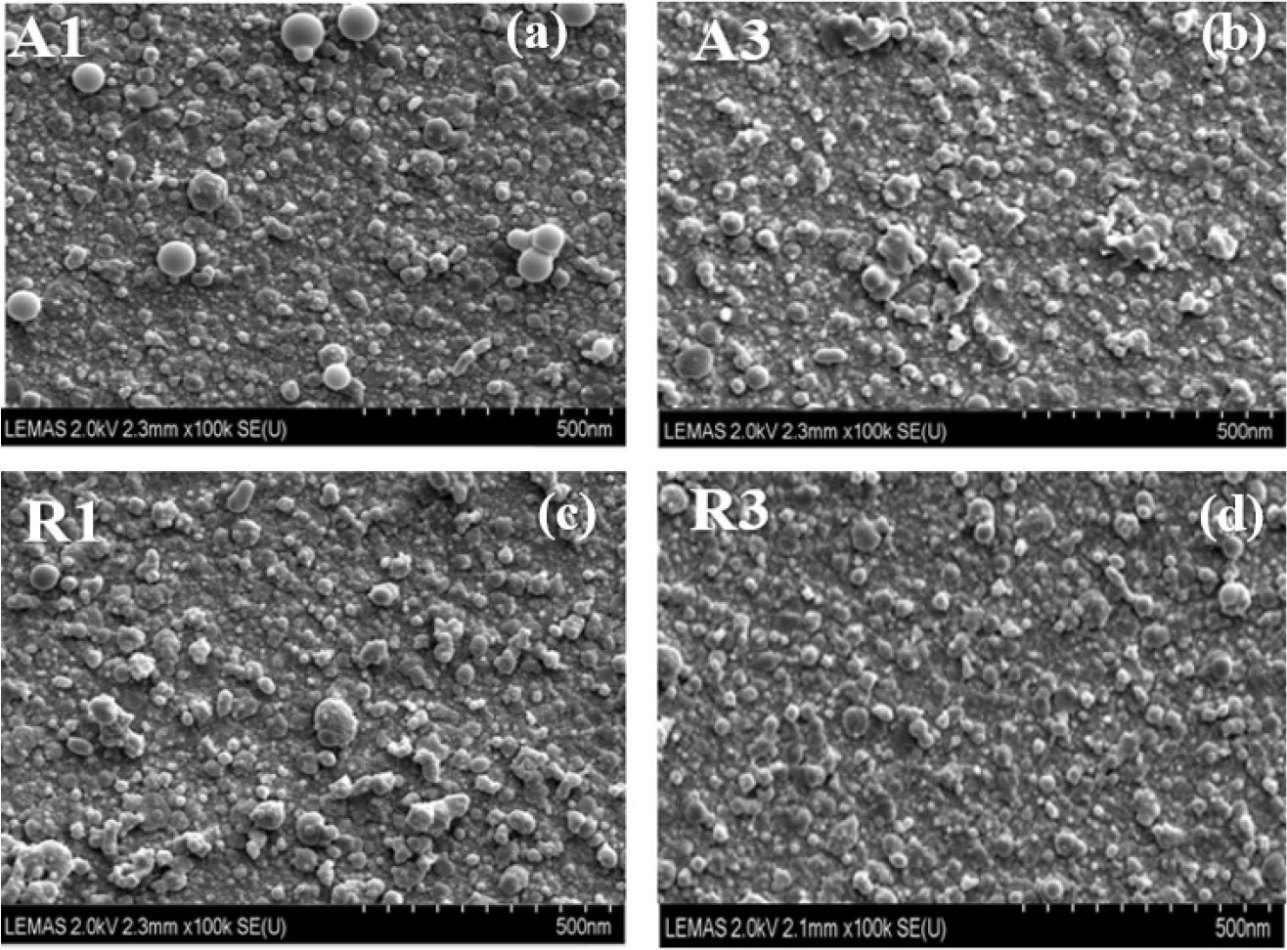
Surface morphologies of TiO_2_ nanoparticulate thin films deposited onto silica substrate by fs-PLD using a scanning emission microscopy (SEM) at various substrate temperatures with 1,280 × 960 pixels and image size of 1,267 nm × 950 nm: anatase thin films: (**a**) [A1 (25 °C)], (**b**) [A3 (700 °C)] and rutile thin films: (**c**) [R1 (25 °C)] (**d**) [R3 (700 °C)].

Prior to fabrication of the TiO_2_ nanoparticulate thin films at various temperatures, optimisation of process parameters such as fs-laser fluence, background oxygen gas pressure, and target-substrate distance were initially determined and implemented. Following this, the optimal processing parameters of the laser fluence for ablation threshold of the TiO_2_ targets, target-substrate distance, and chamber background oxygen pressure were also found to be 3.34 J/cm^2^, 80 mm, and 1mTorr. The SEM images of the anatase and rutile TiO_2_ nanoparticulate thin films as-deposited on silica substrate prepared at various substrate temperatures ranging from room temperature (25 °C) to 700 °C are shown in Fig. 1. An ImageJ software was employed to measure the nanoparticles size distribution statistically using the SEM images shown in Fig. 1.

**Figure 2:**
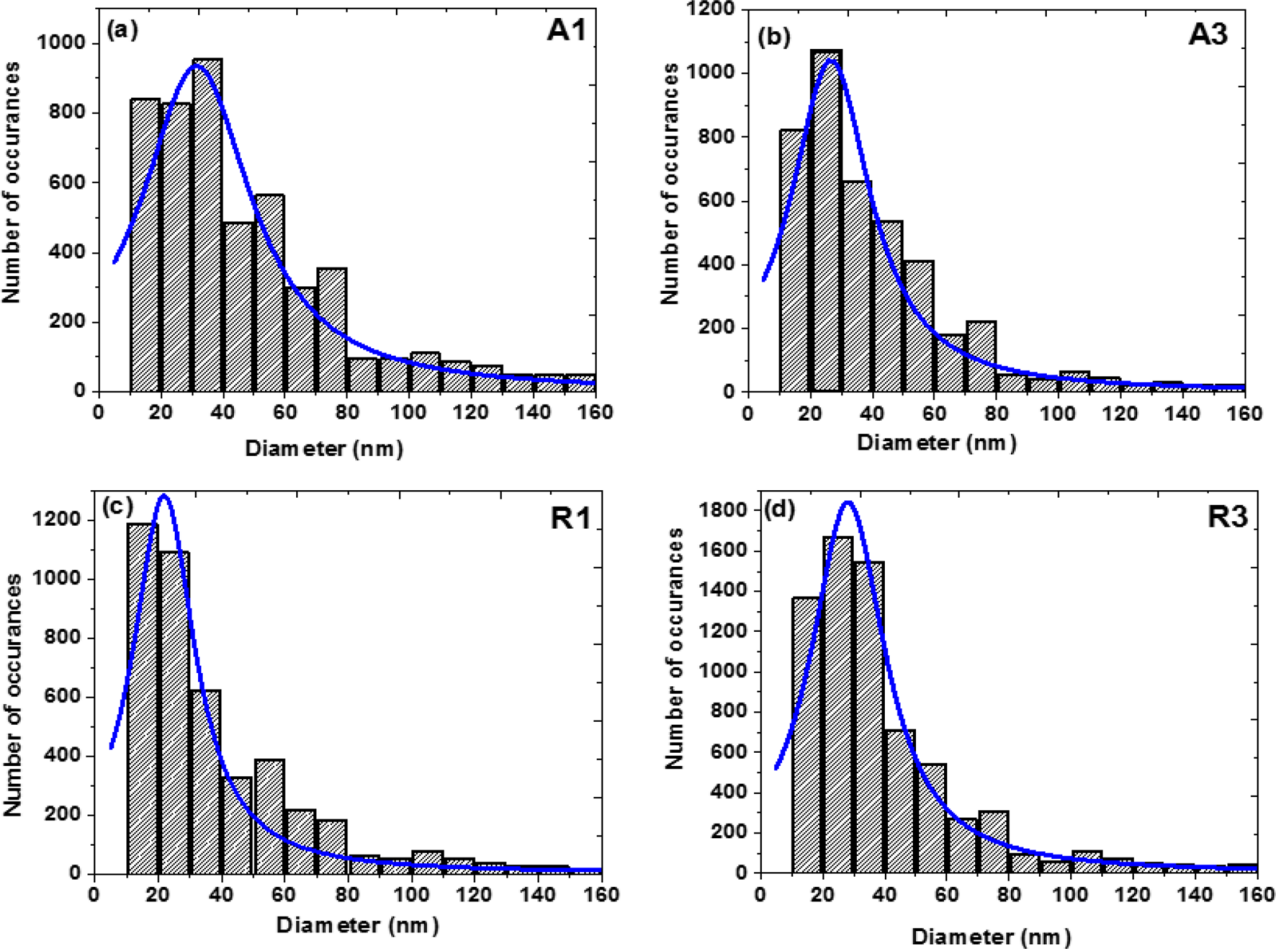
Nanoparticles size distribution histograms obtained from the SEM Images on a grid of 800 × 600 pixels and 100,000 × magnification [1 pixel = 1 nm] shown in Fig. 1 for samples fabricated at different substrate temperature (**a**) A1 (25 °C), (**b**) A3 (700 °C), (**c**) R1 (25 °C) and (**d**) R3(700 °C).

Figure 2a–d illustrates the difference in nanoparticles size distribution (via a histogram) fitted with Lorentzian curve of the SEM image analysis for samples A1, A3, R1, and R3 with each pixel size corresponds to ~ 1 nm. It is important to mention that smaller SEM image magnification was used to determine the particles size such that enough nanoparticles with good resolution can be tested. For anatase samples synthesized (Fig. 1a,b), about ~ 63.9%, ~ 66.8% and ~ 74.2% of nanoparticles have particle sizes of less than 35 nm at 25 °C, 400 °C and 700 °C, respectively. This shows that raising the substrate temperature does not have any significantly influence on the nanoparticle size distribution. For instance, Li et. al.^35^ synthesised doped and undoped TiO_2_ nanoparticles deposited on stainless steel mesh by utilising a metalorganic chemical vapour deposition technique. It was demonstrated that the anatase to rutile phase transformation occurred at a substrate temperature of 700 °C along with an observable increase in the nanoparticles size distribution. On the other hand, the nanoparticulate size distribution histograms of our rutile thin films indicate that ~ 69.3%, ~ 70.0%, and ~ 72.5% of the nanoparticulate film have particle sizes under 35 nm for samples fabricated at 25 °C, 400 °C and 700 °C. The size distribution histograms in Fig. 2 reveal slight increasing in number of rutile nanoparticle sizes lower than 35 nm in relation to increasing substrate temperature. This shows that the influence of substrate temperature on particle size is not significant. Moreover, the nanoparticle sizes remain relatively uniform and homogeneously distributed across the entire substrates for both TiO_2_ nanoparticulate thin films. These results demonstrate that a fs-PLD is more efficient in producing smaller TiO_2_ nanoparticle size than nanosecond laser PLD. For instance, Walczak et al.^26^ grown TiO_2_ nanocrystalline thin films onto silicon substrate by employing nanosecond laser PLD. They noticed that the sample deposited at room temperature showed a small density of aggregation with particles sizes below 500 nm. Whereas those samples fabricated at substrate temperatures at 500 °C and above increased in clusters and aggregation of the nanoparticles with average nanoparticle size of nearly 150 nm at all temperatures. In addition to this, Sanz et al.^28^ used TiO_2_ sintered rutile target to fabricate TiO_2_ thin films onto silicon substrate by utilising three different nanosecond Q-switched Nd:YAG lasers. The aim of this work was to study the influence of laser irradiation wavelength, the substrate temperature, and the presence of oxygen as a background gas pressure. However, the size of microparticulates grown on the silicon substrate ranged from 200 to 2000 nm for substrate temperature spanned from 500 to 700 °C.

**Figure 3:**
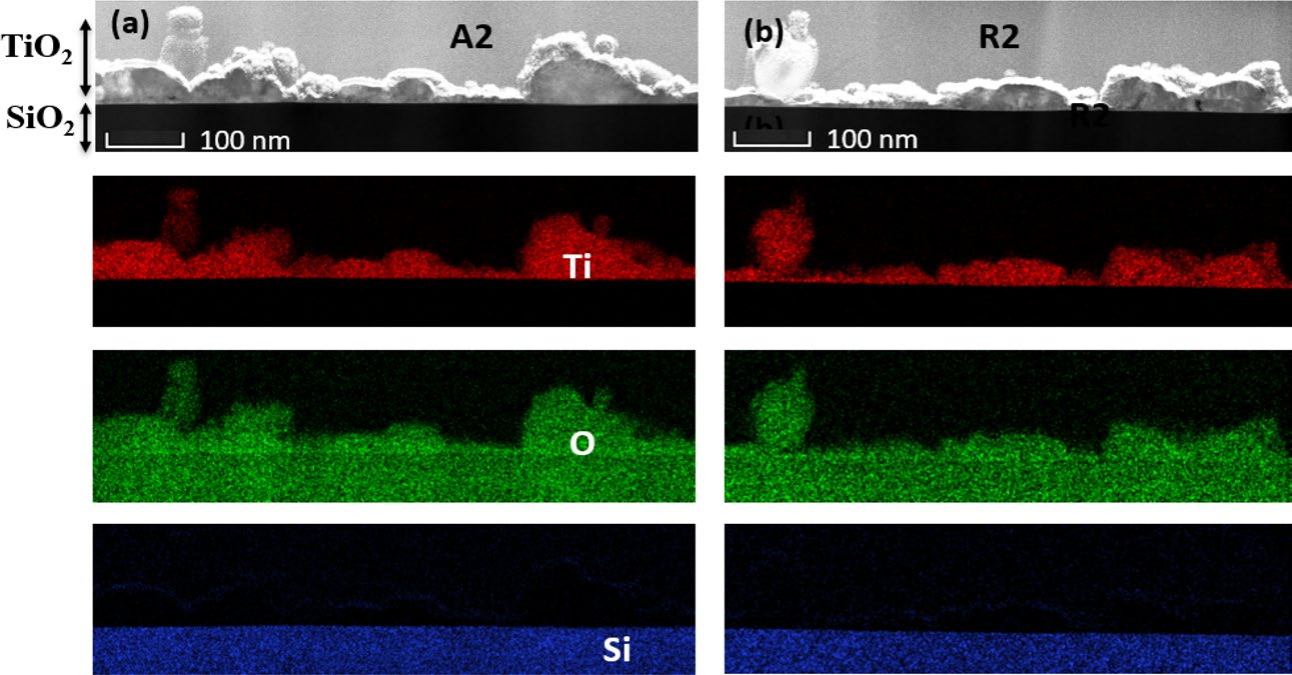
The EDX chemical mapping of the cross-section STEM image for TiO_2_ nanoparticulate thin films indicating that Ti and O elements are evenly distributed on the SiO_2_ substrate (lowermost layer): (**a**) anatase and (**b**) rutile film deposited at 400 °C.

An integrated DualBeam of FIB and FESEM instrument was employed to mill the cross section of the TiO_2_ nanoparticulate thin films deposited on silica substrate for TEM analysis. The TEM–EDX mappings analysis were also carried out to determine elemental compositions as shown in Fig. 3. These elemental species comprise of Ti, and O are uniformly distributed in the nanoparticulate thin films for samples A2 (Fig. 3a) and R2 (Fig. 3b) as well as other samples not shown here. Furthermore, the plain-view of the TEM cross-sections reveal that the films thicknesses are quite non-uniform, which range from 30 to 125 nm.

**Figure 4:**
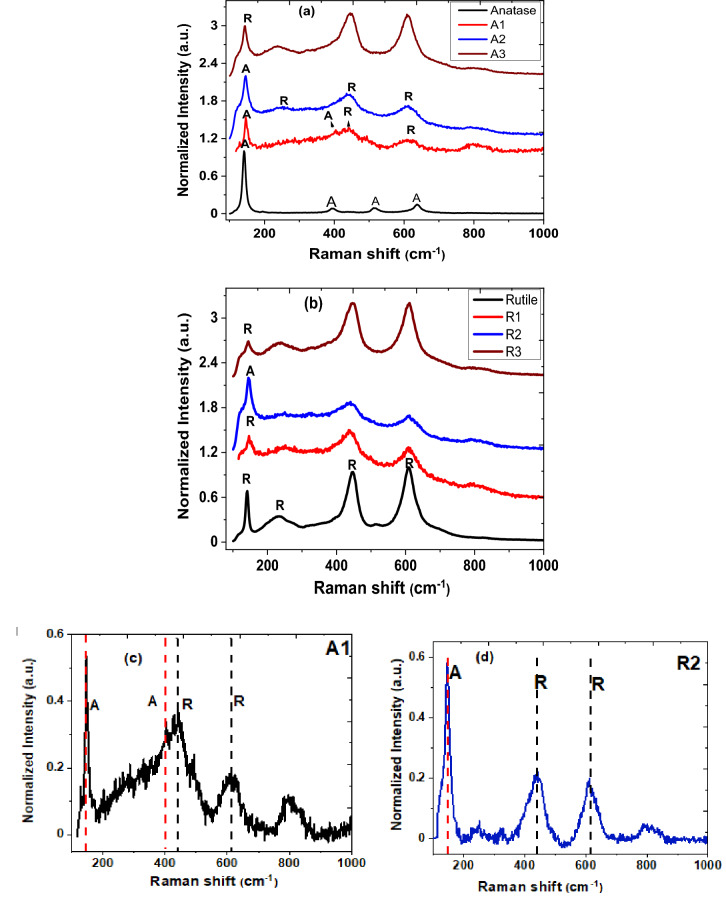
Raman scattering spectra of TiO_2_ targets and TiO_2_ nanoparticulate thin film deposited on silica substrate at various temperatures (**a**) anatase [A1 (25 °C), A2 (400 °C) and A3 (700 °C)] and (**b**) rutile [R1 (25 °C), R2 (400 °C) and R3 (700 °C)]). While (**c**) A1(25 °C) and (**d**) R2 (400 °C) correspond to mixed-phase of anatase and rutile shown enlarged.

The powdered samples of rutile-target, anatase-target, and nanoparticulate thin films fabricated were initially analysed via Raman spectroscopy for structural identification and distinguishing between various polymorph phases of the TiO_2_. Figure 4a,b represents a plot of Raman spectra of powdered samples and their corresponding nanoparticulate thin films deposited at various substrate temperatures. For convenience, A and R are denoted as anatase and rutile peaks. In Fig. 4a, the anatase target material has Raman vibrational bands centred at 144, 197, 398, 513, and 638 cm^−1^, respectively, which correspond to Raman active modes of E_g_, E_g_, B_1g_, A_1g_, and E_g_^36^. However, TiO_2_ nanoparticulate thin film fabricated at room temperature [A1 (25 °C)] exhibits anatase/rutile mixed-phase with Raman vibration modes peaked at 144 (A), 398 (A), 443 (R), 610 (R) and 803 cm^−1^ depicted in Fig. 4c for clarity. The weak broad band centred appear around 800 cm^−1^ is assigned to the silica substrate and Si–O stretching band^37,38^. The anatase nanoparticulate thin film deposited at 400 °C exhibit mixed-phases of anatase and rutile with Raman frequencies centred at 144 (A), 248 (R), 445 (R), and 612 (R) cm^−1^ respectively. The prominently intense peak at 144 cm^−1^ shown in the Raman scattering spectrum confirms the presence of anatase phase while the other peaks are consistent with the rutile polymorph reported^39^. Furthermore, thin films grown from anatase target at a substrate temperature of 700 °C undergoes a complete phase transformation to rutile polymorph. The Raman modes exhibit characteristics of the rutile peaks in agreement with Raman vibration modes previously reported by Byrne et al.^40^. In Fig. 4b, Raman spectra of the rutile target and the thin films prepared at different substrate temperatures are presented. The TiO_2_ rutile target reveals four vibrational bands at 143, 238 (second order effect), 445, 612, and 824 cm^−1^ which agree with the Raman active modes B_1g_, E_g_, A_1g_, and B_2g_^41,42^. The rutile nanoparticulate thin films were also investigated by using Raman spectroscopy. The Raman vibration bands correlate with rutile polymorph peak. Conversely, the rutile thin film fabricated at a substrate temperature of 400 °C reveals a high intensity at the lower frequency mode, 144 cm^−1^ (shown as enlarged in Fig. 4d), in the Raman spectrum. This indicates the presence of anatase phase in the rutile nanoparticle-assembled thin film and such a high intensity frequency is not observed in the rutile polymorph.

**Figure 5:**
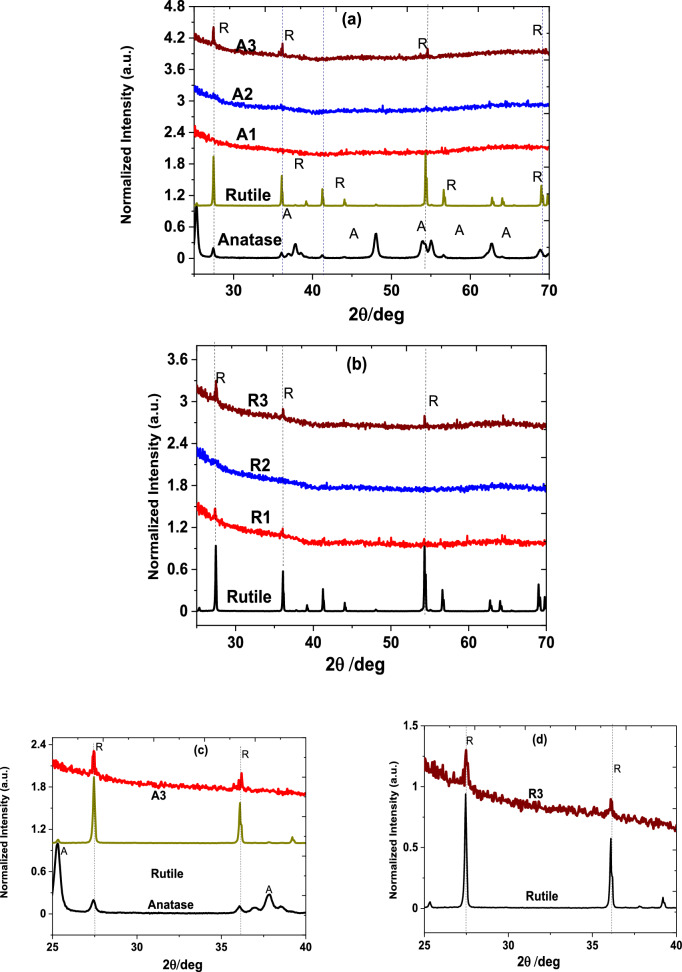
X-ray diffraction patterns of anatase and/or rutile nanoparticles and their corresponding nanoparticles-assembled thin films fabricated by fs-PLD technique at various substrate temperatures: (**a**) anatase [anatase and rutile targets, A1 (25 °C), A2 (400 °C) and A3 (700 °C)]), (**b**) rutile [rutile target, R1 (25 °C), R2 (400 °C) and R3 (700 °C)]). While (**c**) A3 (700 °C) and (**d**) R3 (700 °C) for 2θ range from 25 to 40° shows XRD pattern enlarged clearly demonstrating with the peaks at 2θ = 27.5° and 36.1°.

The XRD patterns of the anatase and rutile TiO_2_ target materials and their corresponding nanoparticle-assembled thin films were also analysed, as this technique can provide structural parameters of crystalline materials’. Figure 5a shows the XRD patterns of the anatase and rutile TiO_2_ targets along with the anatase nanoparticle-assembled films deposited on silica substrate temperature range from 25 to 700 °C. The TiO_2_ anatase target exhibits crystalline structure with diffraction peaks at 2θ of 25.24° (101), 37.87° (103), 53.88° (105) and 62.76° (213), respectively, and the International Centre for Diffraction Data (ICDD) card #00-001-0562. On the other hand, the XRD patterns of the TiO_2_ rutile target are quite intense and narrow with the peak intensities occur at 27.50 (111), 36.04 (101), 41.31 (111), 54.28 (211), and 69.01 (301). The rutile diffraction patterns can be ascribed to ICDD card #00-001-1292 and 01-072-4813. It is worth mentioning that no other TiO_2_ polymorph was identified in the target anatase and/or rutile patterns. Nonetheless, the anatase nanoparticulate thin films fabricated at 25 °C and 400 °C also reveal no XRD patterns. This could be because the particles deposited on the silica substrate are too thin, henceforth, XRD instrument could not detect the existing of anatase and rutile or mixed phase as observed in the Raman spectroscopy technique. But the sample deposited at 700 °C (A3) reveals three weak crystalline peaks centred at 2θ of 27.56 (110)°, 36.05 (633)°, and 51.71 (220)°, respectively.

A higher substrate temperature utilising to deposit anatase enables rutile phase transformation. According to HighScore database, these crystalline peaks observed in sample A3 agree with TiO_2_ (rutile), titanium silicate (TiO_2_–SiO_2_), and titanium silicon oxide (Ti_0.04_SiO_0.96_O_2_). They also have ICDD diffraction patterns number of #01-075-6234, 00-043-0055, and 04-006-0515. Such phase transformation of anatase to rutile depends on the thermodynamic kinetics and alters the optical properties as well the operation of devices. Alternatively, Fig. 5b illustrates the XRD patterns of target rutile and its corresponding thin films. The weak diffraction patterns of the rutile nanoparticle-assembled thin films peaked at 2θ of 27.50°, 36.05° and 51.71° match with the target rutile peak. Henceforth, the presence of this diffraction peak indicates that the rutile TiO_2_ deposited at high substrate temperature remains in rutile polymorph without undergoing phase transition. In addition, Fig. 5c, d illustrates the enlarged X-ray diffraction patterns of samples A3 and R3 compared with anatase and rutile targets for 2θ range from 25° to 40°, which clearly reveal rutile diffraction patterns peaked at 2θ = 27.5° and 36.1°.

### Optical properties

The optical transmittance spectra of as-deposited thin films of anatase and rutile were measured using Perkin Elmer Lambda 905 UV–VIS–NIR Spectrophotometer. These films exhibit good transmission in the spectral range of 250–2,000 nm wavelength. While the reflectance spectra for both samples were recorded with the same Spectrophotometer in a wavelength range from 300 to 1,100 nm. The TiO_2_ anatase and rutile nanoparticulate thin films increase transmittance with higher substrate temperature in the visible and near-infrared spectra range. However, there is strong absorption in the ultraviolet (uv) and low visible wavelength regions, which is characterised by a sharp drop in optical transmittance and increase reflectance. This can be ascribed to fundamental absorption of TiO_2_ nanoparticulate thin film layer owing to the transition’s electron from the valence to conduction bands^43^. This explains why anatase and rutile TiO_2_ thin film is attractive for photocatalytic and solar cells applications^17,18,43^. Also, the transmittance at a given temperature for both anatase and rutile phase show similar properties. But the transmittance absorption edge is sensitive to change in substrate temperature, which is shifted to long uv and low visible wavelengths as the substrate temperature increases. For instance, anatase deposited thin films at various substrate temperatures have absorption edge at ~ 318 (A1), 360 (A2), and 351 (A3) nm. Similarly, the rutile samples R1, R2 and R3 have transmittance absorption edge at ~ 328, 357 and 395 mm.

**Figure 6:**
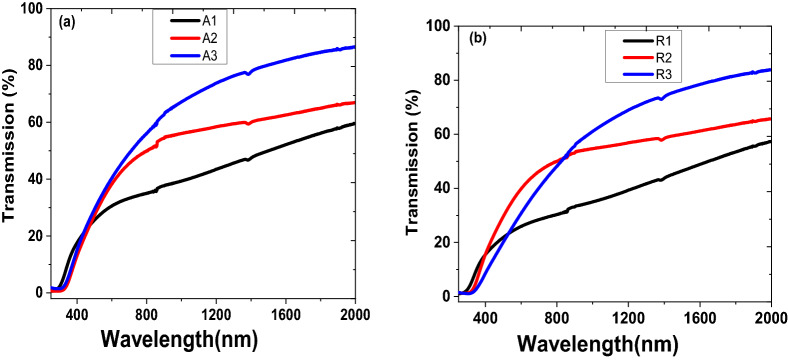
Transmittance of TiO_2_ nanoparticulate thin films deposited on silica substrate at temperature range from 25 to 700 °C; (**a**) anatase and (**b**) rutile.

**Figure 7:**
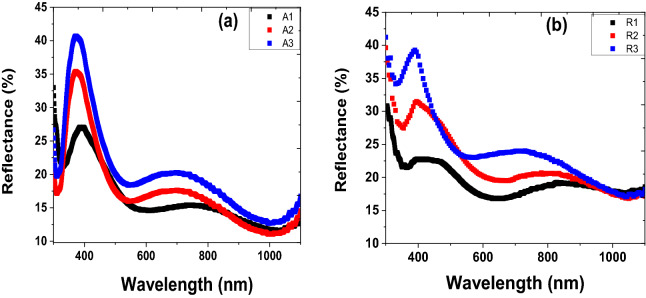
Reflectance spectra of the TiO_2_ thin films (**a**) anatase and (**b**) rutile.

The optical absorption coefficient, α, spectrum was obtained from the transmittance and reflectance spectra shown in Figs. 6 and 7 by employing lambert’s law relationship^44^:

1 $$ T = \left( {1 - R} \right)^{2} e^{ - \alpha t} $$

where T is the transmission, R is the reflectance, and t is the thickness of the film. It worth mentioning that the maximum reflectance of the samples is less than ~ 40% in the 340–420 nm spectral range. Then, Eq. (1) can be expressed as:

2 $$ \alpha = \frac{1}{t}{\ln}\left( {\frac{{\left( {1 - R} \right)^{2} }}{T}} \right) $$

The optical bandgap of the nanoparticulate thin films on the silica substrate is estimated by utilising the relationship between α and energy of incident photons $\left( {h\upsilon } \right)$ known as the Tauc relation^44,45^;

3 $$ \left( {\alpha h\upsilon } \right) = k\left( {h\upsilon - E_{g} } \right)^{n} $$

where h is the planck’s constant, υ is frequency, *E*_g_ is the optical bandgap, *k* is a proportionality constant, *n is* probability factor that takes value of 1/2 (direct allowed), 1 (indirect allowed), 3/2(direct forbidden), or 2 (indirect forbidden) depending on the transition responsible for the absorption, respectively. All the probability power factors, *n*, in Eq. (2) were tested to plot $$\left( {\alpha h\upsilon } \right)^{\frac{1}{n}}$$ versus the incident photon energy $$\left( {h\upsilon } \right)$$ for the TiO_2_ anatase and rutile nanoparticulate thin films. Nonetheless, it was found that $$n = {\raise0.7ex\hbox{$1$} \!\mathord{\left/ {\vphantom {1 2}}\right.\kern-\nulldelimiterspace} \!\lower0.7ex\hbox{$2$}}$$ for direct allowed shows evidence of a perfect fit. Henceforth, Tauc relation of $$\left( {\alpha h\upsilon } \right)^{2} = k\left( {h\upsilon - E_{g} } \right)$$ was employed to determine the optical bandgap spectra shown in Fig. 7.

**Figure 8:**
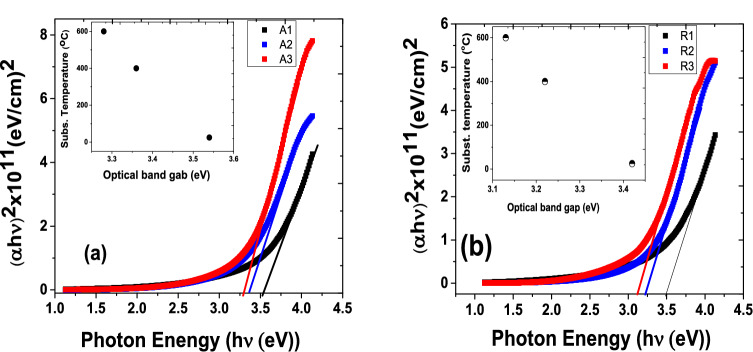
Optical bandgap energies of TiO_2_ nanoparticle-assembled thin films (**a**) anatase and (**b**) rutile.

An inset in Fig. 8a shows substrate temperature versus optical bandgap of the anatase nanoparticulate thin films. The optical bandgap increases slight from 3.3 to 3.5 eV with decreasing substrate temperature from 700° to 25°. Similarly, the inset in Fig. 8b represents rutile thin films optical bandgap energies versus substrate temperature, which range from 3.1 to 3.4 eV. It had been reported that an increase in substrate temperature result in decreasing optical bandgap^45–49^, which is attributed to increase in crystalline size as already discussed in Fig. 2. Moreover, the decrease in optical bandgap in the anatase-target produced thin film is also ascribed to the phase transition into rutile^46^. In addition, an increased in substrate temperature promotes electron into the conduction band and then generate holes in the valence band, there by producing electron at the tail end of the UV spectrum ^50^.

### Electrical properties measurements

Hall Effect measurements of the nanoparticulate thin films prepared on ITO were performed through Van der Pauw method with an excitation current of 10 mA. The excitation current was applied constantly perpendicular to the thin films. Initially, the sheet resistance of each thin film was obtained by measuring the Hall voltage across the Hall field and later used to compute other important parameters listed on Table 1. Table 1 exhibits electrical properties such as sheet resistance (R_S_), free carrier concentrations (n), doping concentrations (D_C_), mobility (µ), Hall co-efficient (R_H_) and resistivity (ρ) of rutile (R) and anatase (A) target prepared thin film on ITO substrate. It can be seen that the resistivity of the as-deposited anatase nanoparticle-assembled thin films on ITO substrate decreases with increasing substrate temperature. Such decrease in the resistivity is a clear indication that the conductivity increases with substrate temperature. As a result, this trend is attributed to increase of carrier mobility and changes in the stoichiometric property of the TiO_2_ thin film^51,52^. Besides, the decrease in resistivity can also be ascribed to increase in crystallite size as shown in Fig. 2 as well as decreased in crystallite boundaries and the phase transformation of anatase to rutile, respectively. In the case of the rutile nanoparticle-assembled on ITO, the resistivity of sample fabricated at 400 °C is found to be 138 × 10^–6^ Ω cm. This could be attributed to formation of anatase–rutile mixed-phase, which has already been discussed in Fig. 3b, d. However, we are unable to measure the sheet resistances and resistivity’s for those samples prepared at substrate temperature of 25 °C and 700 °C.

**Table 1:** Hall Effect measurements parameters of the rutile (R2i) and anatase (A2i) thin films on ITO substrates where i = 1, 2, 3 corresponds to substrate temperature of 25, 400, and 700 °C.

Sample ID	Sheet Resistance (R_s_) (Ω/square)	Film Thickness d (10^–7^ cm)	Hall coefficient, R_H_ (cm^3^/C)	Mobility (µ) (cm^2^/V s)	Carrier Conc. (n) (10^21^ (1/cm^3^))	Carrier type	Doping Conc. (D_c_) (10^16^ 1/cm^2^)	Resistivity (ρ) (× 10^–6^ Ω cm)
R22	11.3	122.29	2.97 × 10^–3^	32.51	2.24	N	1.70	138
A21	11.7	98.09	2.43 × 10^–3^	27.38	2.57	N	1.95	114.0
A22	11.8	71.39	2.64 × 10^–3^	29.41	2.35	N	1.80	84.3
A23	12.5	59.7	2.72 × 10^–3^	28.53	2.30	N	1.74	74.0

## Conclusion

In conclusion, fs-PLD technique has been successfully used to deposit TiO_2_ anatase and rutile nanoparticulate thin films on silica and ITO substrates at different temperatures in the range of 25–700 °C. Raman spectral measurements of both samples deposited at substrate temperature of 400 °C confirmed mixed phase TiO_2_ nanoparticle-assembled thin films. However, anatase target undergoes a complete phase transformation to rutile at substrate temperature of 700 °C. The optical transmittance of anatase and rutile TiO_2_ thin films deposited onto silica substrate increased in optical absorption region with increasing the substrate temperature. This leads to decrease in optical bandgaps of the nanoparticle-assembled films. The electrical properties of the TiO_2_ nanoparticle-assembled on ITO substrates were also studied via Hall Effect measurements. The resistivity’s of the anatase thin films on ITO were observed to be dependent on the substrate temperature, which decrease with increasing in substrate temperature. The resistivity of rutile sample deposition on the ITO substrate at a temperature of 400 °C was measured to be 138 × 10^–6^ Ω.cm, which is ascribed to the presence of anatase–rutile TiO_2_ mixed-phase. Nevertheless, we were unable to measure the sheet resistance of samples fabricated at 25 °C and 700 °C. Therefore, fs-PLD technique has been used to synthesis and control TiO_2_ nanostructure thin films, polymorph-phases, optical and electrical properties with huge potential for photovoltaics, antireflection coatings, solar cells, and photo-catalysis applications.

## Experimental section

### Thin film fabrication

Commercially available TiO_2_ powders of rutile (> 99.9%) and anatase (99.9%) form were obtained from Santa Cruz Biotechnology and Sigma Aldrich (powder < 5 μm). Both metal-based powder samples were pressed into pallet of 25 mm diameter and 4 mm thickness by using a Speca press set at 17 tonne loads for approximately 20 min. The TiO_2_ target and silica substrate of dimensions 20 mm × 30 mm × 1.1 mm were mounted into their respective holders with the substrate positioned parallel to the target. The distance between target and substrate was maintained at 80 mm apart. Besides, the silica substrates were initially cleaned in an ultrasonic bath with acetone, followed by isopropyl alcohol and then dried with a clean lens tissue. The target was rastered and rotated at 40 rpm with the substrate rotation rate of 20 rpm. Prior to the depositions, the fs-PLD vacuum chamber was evacuated to a base pressure of ~ 10^−5^ mTorr, and subsequently injected with high-purity process oxygen gas to adjust the chamber pressure to 1 mTorr. A femtosecond laser equipped with the following specifications was used: wavelength = 800 nm, pulse duration = 100 fs, pulse repetition rate = 1 kHz. The target TiO_2_ samples were ablated with a laser beam spot diameter of ~ 60 µm and a laser fluence of 3.34 J/cm^2^ at 60° angle of incidence. The plasma plume consists of nanoparticulate generated from the target is directed perpendicular to the substrate surface. Furthermore, the substrate heater assembly comprises of a water-cooled nickel/gold-plated copper shroud with silicon carbide heating elements. A type K thermocouple provides feedback to the Eurotherm controller, which is inserted through an opening at the top of the heater assembly to monitor the temperature. Once the substrate is mounted in its respective holder, the temperature reading is provided via closed loop temperature feedback to the PID control loop of the Eurotherm controller in conjunction with the heater DC power supply monitors, which provides the substrate temperature calibrated using a pyrometer. Each deposition was limited to 1 h and the substrate temperature was maintained at 25 °C, 400 °C and 700 °C for samples produced from each target material. The samples were denoted as A1 (25 °C), A2 (400 °C), A3 (700 °C) for thin films prepared from the anatase-TiO_2_ target material and R1 (25 °C), R2 (400 °C), R3 (700 °C) for those obtained from the rutile-TiO_2_ target material. In addition, the anatase and rutile powders were also deposited on indium tin oxide (ITO) under the same experimental conditions, which were used for electrical properties measurements.

### Characterisation

The nanoparticulate thin film morphological features was analysed by Carl Zeiss EVO MA15 scanning electron microscopy (SEM). The SEM images obtained have pixel sizes of 1,280 × 960, image size of 1,268 nm × 950 nm, and a scale bar of 500 nm. These SEM images were initially calibrated with ImageJ software such that the dimensions remain same unit as displayed on the scalar bar. Because, an ImageJ software package can be used to identify each isolated particle deposited on the substrate and allows for measured distributions of all particle’s sizes with maximum efficiency. While composition of the nanoparticles was determined using transmission electron microscopy Energy Dispersive X-ray spectroscopy (TEM–EDX) of cross-section prepared using focused ion beam (FIB) (FEI Helios G4 CX DualBeam). The Energy Dispersive X-ray spectroscopy (EDX) was used to map the chemical composition of the samples for identification of elemental distribution within the planar TEM cross-section of nanoparticles-assembled thin film. The phase transitions of the TiO_2_ thin films from anatase to rutile and rutile films were characterised via Philips PANalytical X’pert X-ray diffractometer with Cu-Kα radiation (λ = 1.54060A°), and Raman spectroscopy (Renishaw inVia Raman Microscope) with a Ar-ion laser of wavelength 514.5 nm. The Perkin Elmer Lambda 905 UV–visible–NIR spectrometer was employed to record the optical transmission and reflectance through the thin film, which was used to calculate bandgaps of the films. Furthermore, Hall effect study was performed to measure electrical properties such as sheet resistance (R_S_), free carrier concentrations (n), doping concentrations (D_C_), mobility (µ), Hall co-efficient (R_H_) and resistivity (ρ) of TiO_2_ anatase and rutile thin films deposited on ITO substrate under the same deposition condition as the silica substrate. The four-point probe dc Van der Pauw geometry method was employed to measure sheet resistance/resistivity.

## Data Availability

The data that support the findings of this research work are available from the corresponding author upon reasonable request.
